# Transcriptome Analysis Reveals Olfactory System Expression Characteristics of Aquatic Snakes

**DOI:** 10.3389/fgene.2022.825974

**Published:** 2022-01-25

**Authors:** Zhong-Liang Peng, Wei Wu, Chen-Yang Tang, Jin-Long Ren, Dechun Jiang, Jia-Tang Li

**Affiliations:** ^1^ CAS Key Laboratory of Mountain Ecological Restoration and Bioresource Utilization and Ecological Restoration and Biodiversity Conservation Key Laboratory of Sichuan Province, Chengdu Institute of Biology, Chinese Academy of Sciences, Chengdu, China; ^2^ University of Chinese Academy of Sciences, Beijing, China; ^3^ Southeast Asia Biodiversity Research Institute, Chinese Academy of Sciences, Yezin Nay Pyi Taw, Myanmar

**Keywords:** transcriptome, gene expression pattern, snakes, olfactory adaptation, aquatic adaptation

## Abstract

Animal olfactory systems evolved with changes in habitat to detect odor cues from the environment. The aquatic environment, as a unique habitat, poses a formidable challenge for olfactory perception in animals, since the higher density and viscosity of water. The olfactory system in snakes is highly specialized, thus providing the opportunity to explore the adaptive evolution of such systems to unique habitats. To date, however, few studies have explored the changes in gene expression features in the olfactory systems of aquatic snakes. In this study, we carried out RNA sequencing of 26 olfactory tissue samples (vomeronasal organ and olfactory bulb) from two aquatic and two non-aquatic snake species to explore gene expression changes under the aquatic environment. Weighted gene co-expression network analysis showed significant differences in gene expression profiles between aquatic and non-aquatic habitats. The main olfactory systems of the aquatic and non-aquatic snakes were regulated by different genes. Among these genes, *RELN* may contribute to exploring gene expression changes under the aquatic environment by regulating the formation of inhibitory neurons in the granular cell layer and increasing the separation of neuronal patterns to correctly identify complex chemical information. The high expression of *TRPC2* and *V2R* family genes in the accessory olfactory systems of aquatic snakes should enhance their ability to bind water-soluble odor molecules, and thus obtain more information in hydrophytic habitats. This work provides an important foundation for exploring the olfactory adaptation of snakes in special habitats.

## Introduction

Vertebrates have evolved complex phenotypes to adapt to different ecotopes driven by various selection regimes. The olfactory sensory system has played a crucial role in this process due to its involvement in food acquisition, mate and offspring recognition, predator avoidance, and habitat detection (Nei et al., 2008; Gomez-Diaz et al., 2018). Olfactory systems perform critical chemical communication functions, which are highly dependent on environmental media. Notably, propagation media and molecules differ markedly between terrestrial and aquatic environments (Freitag et al., 1998). Higher density and viscosity of water, coupled with a much lower diffusion speed in comparison to air make olfactory perception difficult underwater environment (Weiss et al., 2021). Therefore, the olfactory systems of animals have evolved to adapt to aquatic habitats. For instance, unlike for terrestrial mammals, in aquatic mammals the vomeronasal system is inactivated and the olfactory nervous system may be lost (Thewissen and Nummela, 2008; Kishida, 2021).

Snakes are a special group of animals that have evolved a suite of complex traits that enable them to survive in a variety of habitats worldwide (Peng et al., 2020; Ren et al., 2022). Their sensory system is unique among vertebrates, with severely degraded visual and acoustic faculties (Christensen et al., 2012; Lillywhite, 2014; Cronin, 2020) but highly developed and specialized olfactory systems. Snakes possess two olfactory systems: i.e., the main olfactory system (MOS), with the olfactory bulb as the core, and the accessory olfactory system (AOS), consisting of the vomeronasal organ and tongue. These two olfactory systems are separated completely and function independently. The MOS is sensitive to airborne molecules and detects volatile chemicals, while the AOS is specifically stimulated by non-volatile and water-soluble macromolecular chemicals, such as pheromones (Wang and Halpern, 1980a; Wang and Halpern, 1980b; Lohman and Smeets, 1993). The highly developed olfaction of snakes, accompanied by the degeneration of other senses, suggests that olfactory system evolution plays an important role in environmental adaptation.

The two olfactory systems of snakes are regulated by different receptors. Odorant receptors (*ORs*) are localized in the MOS and vomeronasal receptors (*V1Rs* and *V2Rs*) play a role in the AOS (Buck and Axel, 1991; Herrada and Dulac, 1997; Mombaerts, 2004). *V1Rs* and class II *ORs* are thought to bind to chemicals in the air, while water-soluble chemical molecules are recognized by *V2Rs* and class I *ORs* (Shi and Zhang, 2007; Nei et al., 2008; Saito et al., 2009). Snakes contain many *OR* and *V2R* genes (Halpern, 2003; Hogan et al., 2021). Compared with non-aquatic snakes, the MOS of sea snakes is degenerated and *OR* genes are rarely expressed, but gigantic mapping of *V2Rs* genes exists (Kishida et al., 2019; Li et al., 2021). While previous studies have focused on receptor copy number variation, the comprehensive expression patterns of the olfactory in aquatic snakes remain to be parsed.

To explore the molecular basis of aquatic adaptation of olfaction in snakes, we performed transcriptome sequencing and weighted gene co-expression network analysis (WGCNA) of 26 samples (main olfactory bulb and vomeronasal organ) from two aquatic and two non-aquatic snake species: i.e., *Hypsiscopus plumbea* and *Opisthotropis zhaoermii* are typical groups of aquatic snakes (Murphy and Voris, 2014; Ren et al., 2017), and *Ahaetulla prasine* and *Pareas menglaensis* perched on bushwood (Amber et al., 2017; Wang et al., 2021), thus the four species are practicable models for exploring olfactory characteristics associated with habitat types. We observed considerable environment-dependent differences in overall expression profiles. In the MOS of aquatic snakes, the unique co-expression network regulated by *RELN* may facilitate the accuracy of chemical information identification to adapt to complex underwater environments. In the AOS of aquatic snakes, the expression of *TRPC2* gene and *V2R* family is associated with the ability to bind water-soluble molecules. These findings contribute to our understanding of the physiological mechanisms underpinning olfactory adaptation to the environment.

## Materials and Methods

### Sample Collection

**FIGURE 1:**
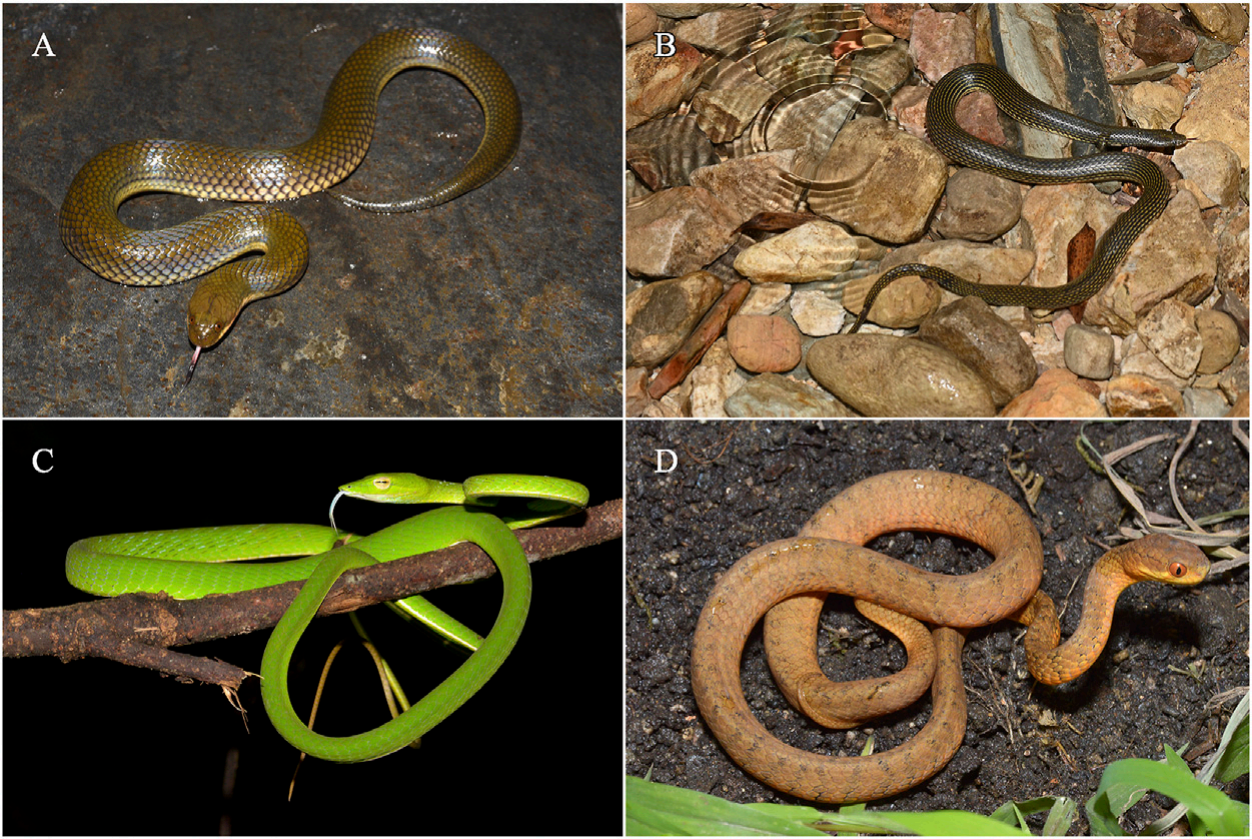
Four species of snakes in the study. **(A)** *Hypsiscopus plumbea* (aquatic). **(B)** *Opisthotropis zhaoermii* (aquatic). **(C)** *Ahaetulla prasina* (non-aquatic). **(D)** *Pareas menglaensis* (non-aquatic). Photos by Jin-Long Ren and Jun-Jie Huang.

This study involved four species: *Ahaetulla prasina*, *Hypsiscopus plumbea*, *Opisthotropis zhaoermii* and *Pareas menglaensis*. We defined two categories of habitat types based on propagation medium: 1) aquatic; 2) non-aquatic. More than 50% of their lifetime is in the water (Sheehy et al., 2015), and they are assumed as aquatic snakes. If not, they are considered as non-aquatic snakes. *H. plumbea* and *O. zhaoermii* were seen commonly in ponds or streams, and *A. prasina* and *P. menglaensis* were discovered in wooded areas frequently (Zhao, 2006). Therefore, *H. plumbea* and *O. zhaoermii* were assumed as aquatic snakes, *A. prasina* and *P. menglaensis* were considered as non-aquatic snakes (Figure 1).

Based on phylogenetic relationships, four distantly related snake species were selected from two different habitats for transcriptome sequencing (Supplementary Figure S1). A total of 13 healthy and adult snakes were collected, including three biological duplications of *A. prasine*, *H. plumbea*, and *O. zhaoermii*, respectively, and four individuals of *P. menglaensis* (Supplementary Table S1). The vomeronasal organ that is an important component of the AOS and the main olfactory bulb that is integral to the MOS of specimens were harvested. The vomeronasal organ and main olfactory bulb tissues were collected using stereo microscope according to Kardong (2012) (Supplementary Figure S2). After sampling, all the tissues were immediately frozen in liquid nitrogen for ten minutes and stored at –80°C prior until RNA isolation.

### RNA Isolation, cDNA Library Construction and mRNA Sequencing

Total RNA was extracted from olfactory tissues using QIAGEN^®^ RNA Mini Kit following the manufacturer’s protocol. After degradation and contamination monitored on 1% agarose gel, RNA purity was checked using the NanoPhotometer^®^ spectrophotometer (IMPLEN, Calabasas, CA, United States). We used the Qubit^®^RNA Assay Kit in Qubit^®^2.0 Flurometer (Life Technologies, Carlsbad, CA, United States) to measure RNA concentration, and used the RNA Nano 6000 Assay Kit of the Agilent Bioanalyzer 2,100 system (Agilent Technologies, Santa Clara, CA, United States) to evaluate the RNA integrity number (RIN). Each sample has extracted 1.5 μg RNA. NEBNext^®^ UltraTM RNA Library Prep Kit for Illumina^®^ (NEB, United States) was used to construct sequencing libraries following the manufacturer’s instruction. Add index code to the attribute sequence of each sample. TruSeq PE Cluster Kit v3-cBot-HS (Illumina) was used to cluster index-coded samples on a cBot Cluster Generation System. RNA sequencing was performed on an Illumina NovaSeq 6,000 platform and 150 bp paired-end reads were generated.

### *De novo* Transcriptome Assembly and Sequence Annotation

The low-quality reads of raw reads data were filtered by using seqtk v1.3-r106, and the remaining were considered as clean reads data. Following preprocessing, the high-quality reads data of 26 samples were employed for *de novo* assembly using Trinity v2.4.0 (Grabherr et al., 2013) with parameters “-group_pairs_distance 230 -min_contig_length 600 -min_glue 4” (Tang et al., 2021), and the resulting sequences were identified as unigenes. TransDecoder v3.0.1 was used to extract the open reading frame (ORF) and predict coding DNA sequence (CDS) (Lee et al., 2015), and BUSCO v3.0.2 (Benchmarking Universal Single-Copy Orthologs) was used to assess the completeness of transcriptome (Simão et al., 2015). We used the BLASTP algorithm for searching longest coding protein genes against the non-redundant SWISS-Port database^1^ (-line 2 -word_size 4 -evalue 1e-5 -top 3) to match the best-retrieved result. Additionally, the same protocol was used against the NCBI non-redundant protein (NR) database and the Kyoto Encyclopedia of Genes and Genomes (KEGG) database. Gene ontology (GO) functional annotation of unigenes was obtained by using an internal Perl script, which is based on SWISS-Prot annotated results. GO annotation results were counted by WEGO 2.0^2^ (Ye et al., 2018).

### Orthologous Genes Identification and Quantification of Gene Expression

BLAST algorithm was used to identify orthologous genes based on amino acid sequences of four species. Sequence alignments were conducted for transcripts between four species (E-value = 1e-5). We obtained reciprocal best hits in each four and retained orthologous genes shared by four species. The TPM (Transcripts Per Million) value was calculated to determine the expression level of each transcript. Gene and isoform abundances were calculated with RSEM (Li and Dewey, 2011).

### Principal Component Analysis and Weighted Gene Co-expression Network Analysis

Principal component analysis (PCA) was applied with the R package FactoMineR (Lê et al., 2008) and factoextra (Kassambara and Mundt, 2017) to gain more insight into the cluster of samples based on expression data of orthologous genes. The co-expression networks of the orthologous genes were constructed using the WGCNA v1.69 package (Langfelder and Horvath, 2008) in R based on the TPM. We calculated expression correlation coefficients of the orthologous genes to select a suitable soft threshold using a scale-free topology model (Wan et al., 2018). The module was defined by setting the minimum number of modules to 150 and the hierarchal clustering dendrogram was cut using the dynamic tree-cutting algorithm (mergeCutHeight = 0.2). Genes were clustered with similar expression patterns into a co-expression module and the module eigengenes (ME) were calculated for each module. The olfactory systems of snakes in two types of habitats were defined as different phenotypes, and we focused on the modules that are most relevant to the traits. Subsequently, we constructed a co-expression network, and visualized using Cytoscape v3.7.2 (Shannon et al., 2003) based on the genes in the corresponding module and identified hub genes of the co-expression network according to the degree method using cytoHubba (Chin et al., 2014).

### Gene Ontology Enrichment Analysis

To understand the potential functions of the module eigengenes, GO enrichment analysis was performed using the R package clusterProfiler v3.14.3 (Yu et al., 2012). The GO annotations with all annotated unigenes were seen as background set to identify significantly enriched GO terms. Enriched terms with *p*-values < 0.05 were considered significant (Liu et al., 2020). And GO enrichment results were completed redundancy removal by REVIGO^3^ (Supek et al., 2011).

## Results

### *De novo* Transcriptome Assembly and Functional Annotation

To compare the olfactory gene expression profiles between aquatic and non-aquatic snakes, we collected 26 samples (main olfactory bulb and vomeronasal organ) from four species of snakes inhabiting two types of habitats. A total of 797.88 million 150-bp paired-end raw reads were generated, and 762.97 million clean reads (95.7%) were retained after quality control. Among the samples, the Q30 scores were >90% and GC content ranged from 42.95% to 46.95% (Supplementary Table S1). The clean reads were assembled using Trinity v2.4.0, generating a total of 207,427 transcripts with an N50 of 3,447 bp for *A. prasina*, 243,737 transcripts with an N50 of 3,512 bp for *H. plumbea*, 217,064 transcripts with an N50 of 3,506 bp for *O. zhaoermii*, and 270,112 transcripts with an N50 of 3,598 bp for *P. menglaensis* (Supplementary Table S2). Based on nucleotide mode and lineage data from vertebrates, BUSCO v3.0.2 (Simão et al., 2015) was used to assess transcriptome assembly, which showed that more than 77% of protein-coding genes were complete and the proportion of fragmented genes was under 10% (Supplementary Table S3).

**FIGURE 2:**
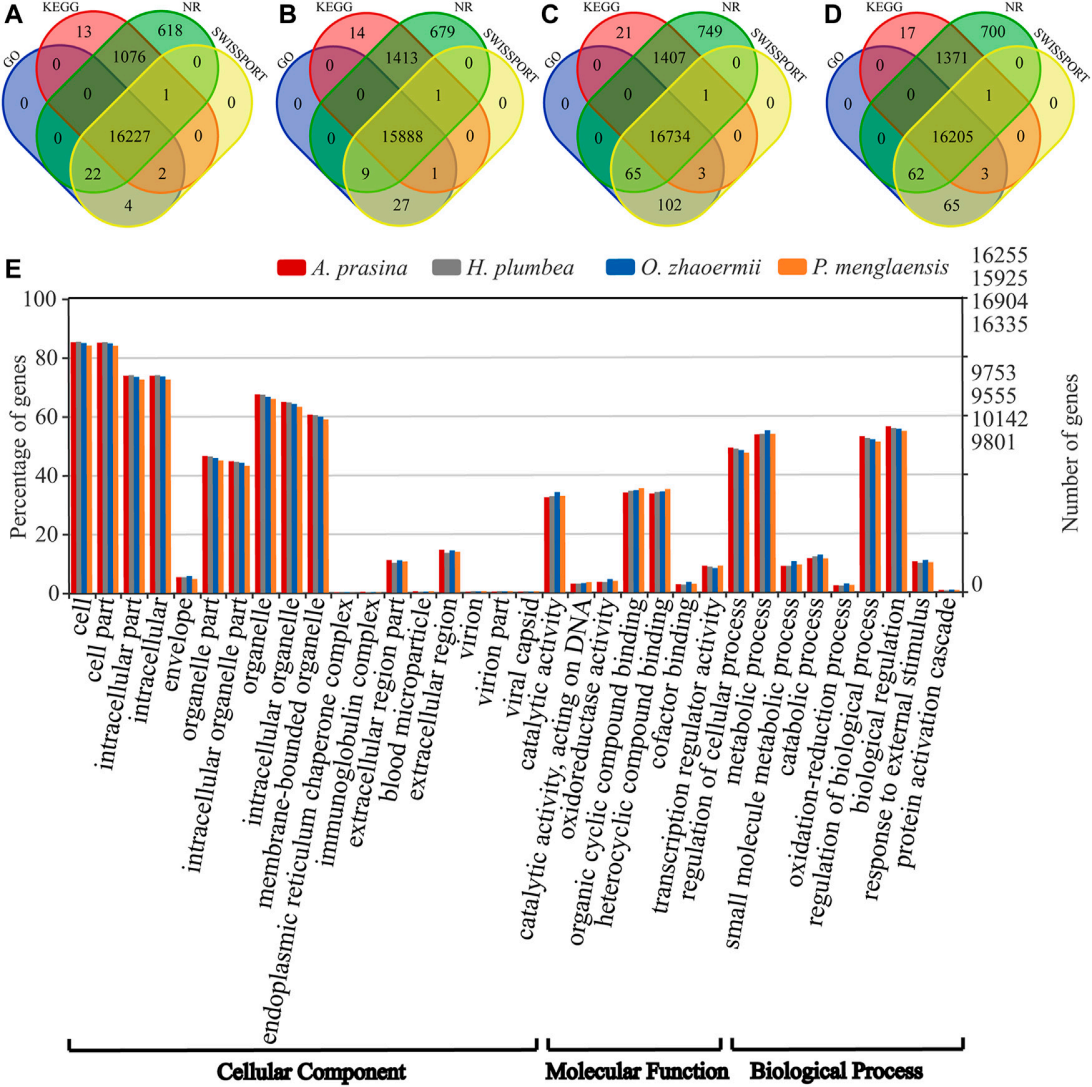
Overview of annotative summary of *de novo* transcriptome assembly. **(A–D)** Venn diagram showing the number of specific and overlapping annotated unigenes between annotated results from four public databases. **(A)** Functional annotation of transcripts in of *A. prasina*. **(B)** Functional annotation of transcripts of *H. plumbea*. **(C)** Functional annotation of transcripts of *O. zhaoermii*. **(D)** Functional annotation of transcripts of *P. menglaensis*. **(E)** Comparison of Gene Ontology (GO) classifications based on *de novo* transcriptome assembly of four species of snakes.

Among the transcripts, 71,526 for *A. prasina*, 74,197 for *H. plumbea*, 77,549 for *O. zhaoermii*, and 94,479 for *P. menglaensis* contained open reading frames (ORFs) that predicted peptides >100 amino acids (aa) in length, including 36,058, 37,947, 38,713 and 44,937 full-length transcripts for predicting protein-coding sequences. After removing redundancy and clustering, 25,839, 27,413, 27,793, and 31,844 of the longest protein-coding transcripts were retained for orthologous gene analysis. The longest protein-coding transcripts were searched against the NR, Gene Ontology (GO), SWISSProt, and Kyoto Encyclopedia of Genes and Genomes (KEGG) databases. Results showed that 17,963 (69.5%) in *A. prasina*, 18,032 (65.8%) in *H. plumbea*, 19,082 (68.7%) in *O. zhaoermii*, and 18,424 (57.9%) in *P. menglaensis* were significantly matched to known genes in the databases (Figures 2A–D). Functional annotation analysis divided these genes into cellular components, molecular functions, and biological process categories, with the annotated GO terms shown in Figure 2E.

### Principal Component Analysis and Weighted Gene Co-expression Network Analysis

**FIGURE 3:**
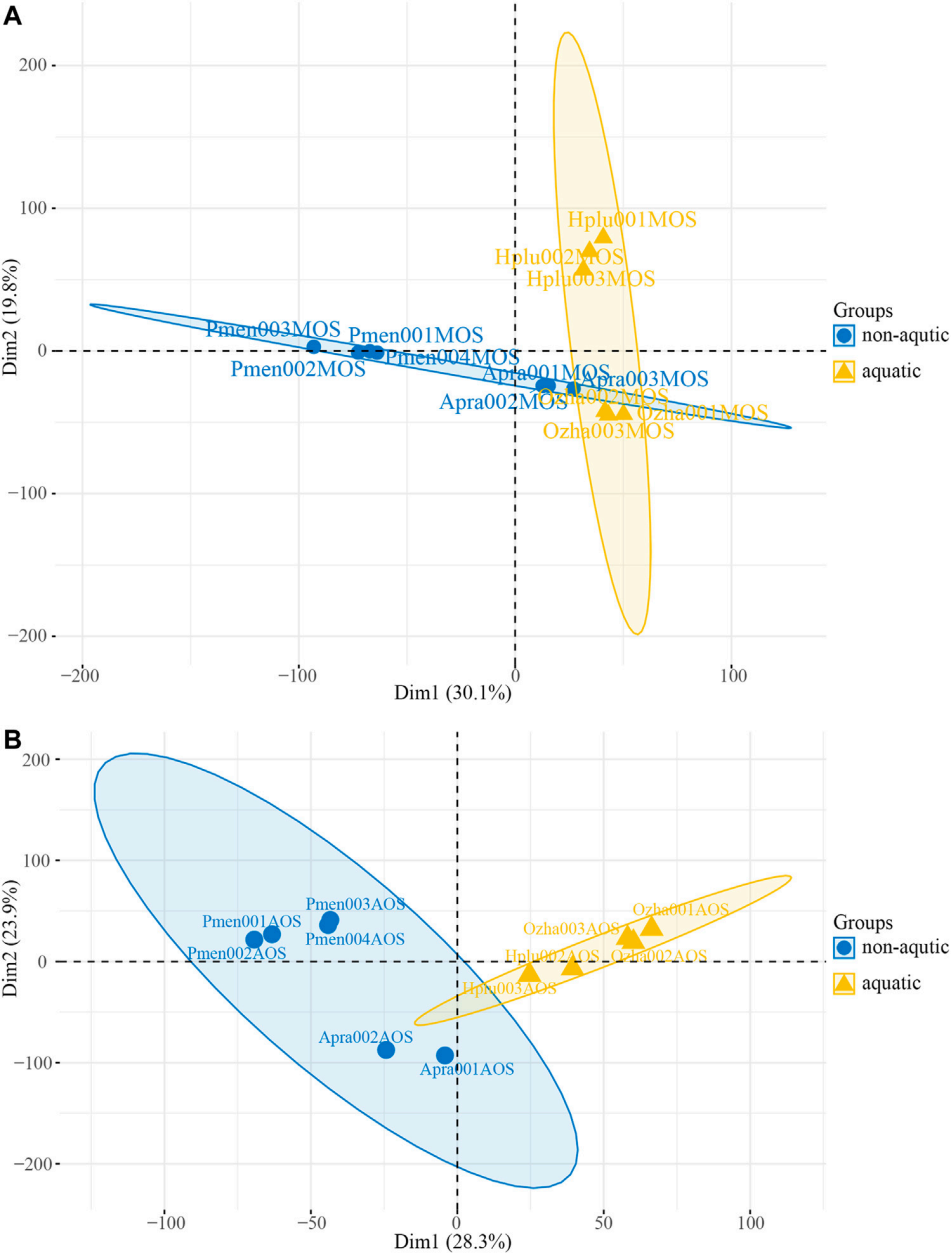
Plot of the principal component analysis (PCA) of four species of snakes. **(A)** PCA plot of main olfactory system (MOS) samples. **(B)** PCA plot of accessory olfactory system (AOS) samples.

PCA was performed based on transcripts per million (TPM) and revealed the presence of two distinct transcriptomic clusters, i.e., MOS and AOS, showing different expression patterns (Supplementary Figure S3A). Regarding to the MOS tissue, aquatic snakes clustered in a group and could be clearly distinguished from *P. menglaensis* (Figure 3A), as well as *A. prasine* (Supplementary Figure S3B). In the AOS data, the aquatic and non-aquatic samples clustered separately and the greatest source of variation was derived from habitat type by PC1 (Figure 3B), indicating considerable differences in olfaction gene expression patterns between aquatic and non-aquatic snakes.

**FIGURE 4:**
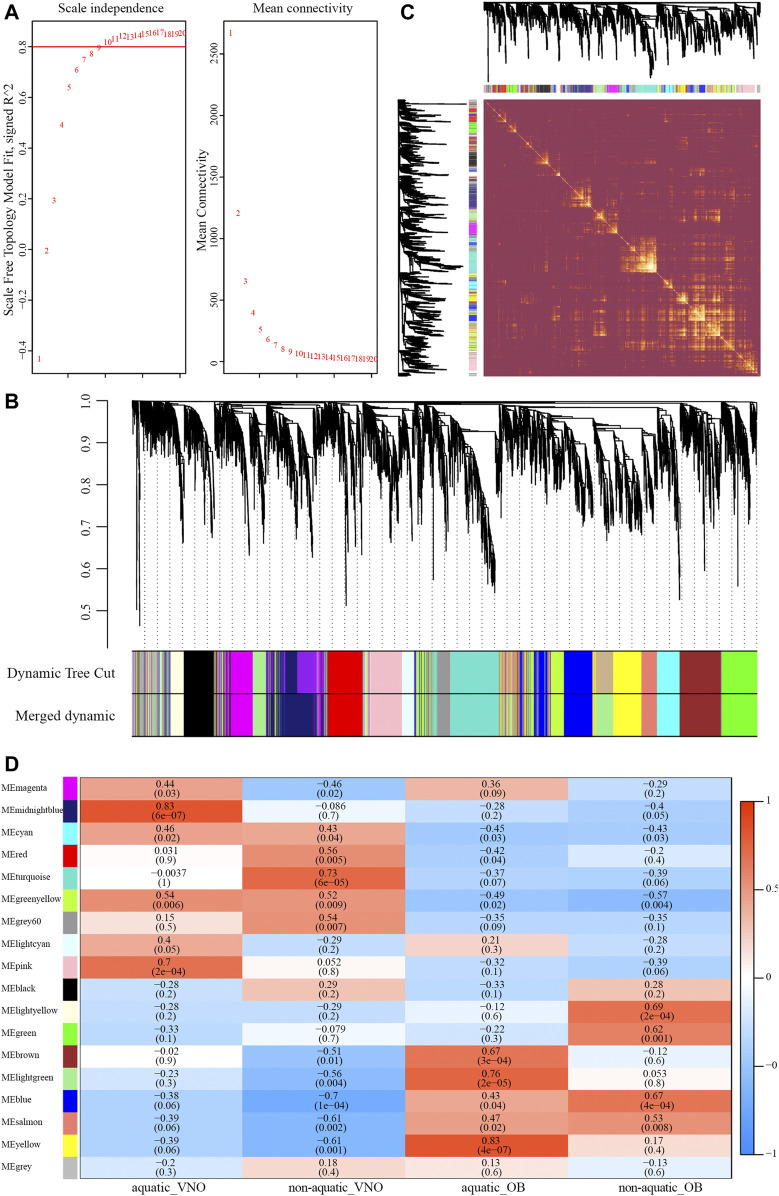
Construction of the weighted gene co-expression network (WGCNA). **(A)** Analysis of soft-thresholding powers based on scale independence (left) and mean connectivity (right). **(B)** The cluster dendrogram of orthologous genes. Each row corresponds to a module eigengene. **(C)** Network heatmap plot of orthologous genes. **(D)** Module-traits relationships identified by WGCNA. The color and the number (above) of each cell indicate the correlation and the numbers in parentheses represent *p*-value.

A total of 8,618 orthologous genes were identified among the four species, which were used to construct weighted gene co-expression networks *via* the WGCNA v1.69 package using the R programming language. A beta power of 12 (Wan et al., 2018) was selected as the soft-thresholding power to ensure a scale-free network (Figure 4A). Strongly co-expressed genes in clusters were screened and assigned to a module, as shown in Figure 4B. The size of the modules depended on the number of genes contained therein, with the turquoise module containing the largest number of genes (861) and the light-yellow module containing the least (212). Genes assigned to the gray and gray60 modules were not co-expressed. The heatmap of the topological overlap matrix of all genes indicated that gene expression was relatively independent between modules (Figure 4C).

To identify modules significantly associated with habitat type, module-trait relationships were analyzed using correlations between module eigengenes and four traits (i.e., aquatic AOS, non-aquatic AOS, aquatic MOS, and non-aquatic MOS) (Figure 4D). The module with the highest correlation coefficient was defined as the habitat-specific module, however, results of enrichment analysis were not significant in the non-aquatic MOS module (light-yellow). Thus, another module that significantly related to the MOS of non-aquatic snakes and olfactory-related items could be significantly enriched was selected. A total of five habitat-specific modules were selected, including the midnight-blue (aquatic AOS, 779 genes), turquoise (non-aquatic AOS, 861 genes), yellow (aquatic MOS, 572 genes), and light-yellow and blue modules (non-aquatic MOS, 212 and 682 genes).

### Differential Expression Patterns in Main Olfactory System Between Aquatic and Non-aquatic Snakes

**FIGURE 5:**
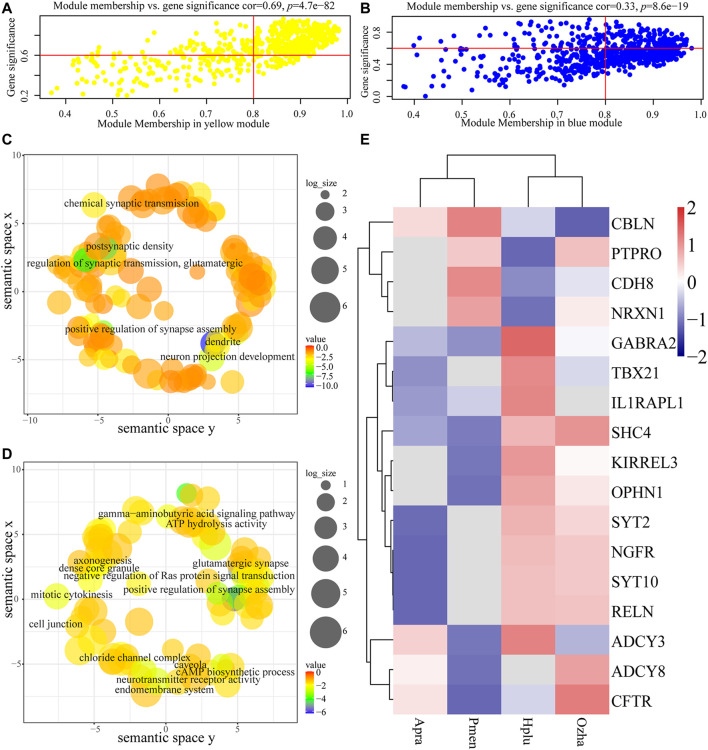
Analysis of the aquatic MOS (yellow) module and the non-aquatic MOS (blue) module. **(A, B)** The *x*-axis is module membership and the *y*-axis stand for gene significance. Gene significance (GS) was calculated to measure the correlation between habitat and expression profiles and module membership (MM) was calculated to measure the correlation between ME and expression profiles. **(A)** Scatterplots of gene significance (GS) for habitat traits versus module membership (MM) in the aquatic MOS module. **(B)** Scatterplots of gene significance (GS) for habitat traits versus module membership (MM) in the non-aquatic MOS module. **(C, D)** Each term was assigned x and y coordinates and more semantically similar GO terms were closer in the plot. The size of the circles indicates the number of child GO terms. **(C)** GO enrichment analysis of genes in the aquatic MOS module was visualized using REVIGO. **(D)** GO enrichment analysis of genes in the non-aquatic MOS module was visualized using REVIGO. **(E)** The expression of olfactory-related genes in the two modules.

For each gene expression profile, gene significance (GS) was calculated to measure the correlation between habitat and expression profiles, and module membership (MM) was calculated to measure the correlation between ME and expression profiles. Scatterplots of GS and MM in the aquatic and non-aquatic MOS-related modules are shown in Figures 5A,B. In the two modules, GS and MM were correlated, indicating that significant habitat-associated genes were important elements of the modules.

Functional enrichment analysis revealed the biological significance of modules. Results showed that genes in the aquatic MOS-related modules (yellow) were mainly involved in the process of neuronal connection (e.g., *KIRREL3*, *OPHN1*, and *SYT2*), including dendrite (GO:0030425), postsynaptic density (GO:0014069), neuron projection development (GO:0031175), and chemical synaptic transmission (GO:0007268) (Figure 5C, Supplementary Table S5). In the non-aquatic MOS-related module (light-yellow), genes were mainly involved in the process of biosynthesis, including Cul3-RING ubiquitin ligase complex (GO:0031463) and cholesterol biosynthetic process (GO:0006695) (Supplementary Table S4). In the non-aquatic MOS-related module (blue), synapse assembly (GO:0051965) and glutamatergic synapse (GO:0098978) were the most abundantly represented GO terms, suggesting that the genes were highly associated with neuronal connection (e.g., *CDH8*, *ADCY3*, *PTPRO*, *NRXN1*, *CBLN2*, *ADCY8*, and *CFTR*) (Figure 5D, Supplementary Table S6). Intramodular connectivity was ranked according to the degree method using the cytoHubba plug-in, and the top 10 genes in each module of interest were used to visualize the specific modules (Supplementary Figure S4B). Among the hub genes, *RELN*, which is involved in the differentiation process of the granular cell layer in main olfactory bulb (Martin-Lopez et al., 2012), was highly expressed in aquatic snakes (Figure 5E).

### Differential Expression Patterns in Accessory Olfactory System Between Aquatic and Non-aquatic Snakes

**FIGURE 6:**
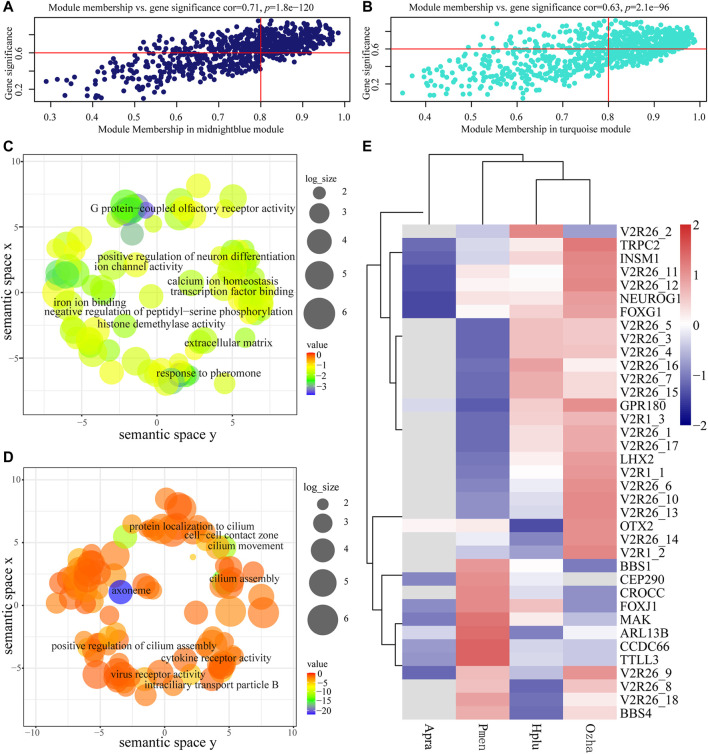
Analysis of the aquatic AOS (midnight-blue) module and the non-aquatic AOS (turquoise) module. **(A)** Scatterplots of gene significance (GS) for habitat traits versus module membership (MM) in the aquatic AOS module. **(B)** Scatterplots of gene significance (GS) for habitat traits versus module membership (MM) in the non-aquatic AOS module. **(C)** GO enrichment analysis of genes in the aquatic AOS module was visualized using REVIGO. **(D)** GO enrichment analysis of genes in the non-aquatic AOS module was visualized using REVIGO. **(E)** The expression of olfactory-related genes in the two modules.

The GS and MM values of AOS-related modules were calculated to measure the correlation between module eigengenes and habitat type, which showed relatively high correlation (Figures 6A,B). Functional enrichment analysis indicated that the genes in the aquatic AOS-related module (midnight-blue) were involved in the process of olfactory signal transduction (e.g., *TRPC2*, *V2R*, and *GPR180*), including response to pheromone (GO:0019236), G protein-coupled olfactory receptor activity (GO:0038022), and neuron differentiation (GO:0030182) (Figure 6C, Supplementary Table S7). In the non-aquatic AOS-related module (turquoise), GO enrichment analysis indicated that the genes mainly participated in the regulation of cilium (e.g., *BBS1*, *BBS4*, and *ARL13B*), including cilium assembly (GO:0060271), cilium (GO:0005929), and motile cilium (GO:0031514) (Figure 6D, Supplementary Table S8).

The top 10 genes with the greatest connectivity were identified as hub genes, which may play roles in olfactory signal transduction (Supplementary Figure S4A). Among these genes, *TRPC2* and *V2R26_10* were identified as olfactory-related genes with involvement in response to pheromone (GO:0019236). A co-expression subnetwork containing *TRPC2* and *V2R26_10* was constructed, which showed that *TRPC2* was co-expressed with 15 receptor genes (14 vomeronasal receptors and *GPR180*) and five neuronal development-related genes (*OTX2*, *LHX2*, *NEUROG1*, *INSM1*, and *FOXG1*). Of which, 11 vomeronasal receptor transcripts, *GRP180*, *LHX2*, *NEUROG1*, *INSM1*, and *FOXG1*, were highly expressed in the vomeronasal organ of aquatic snakes (Figure 6E), suggesting the potential vital role of the subnetwork in adaptations to the water environment.

## Discussion

In animals, olfaction plays an important physiological function by sensing chemical cues in the environment, which provide key information on food, mates, and predators, thereby allowing organisms to make critical behavioral decisions at greater distances (Korsching, 2016). Different environments have different communication media, and it is hypothesized that transition to a new environment can result in olfactory system adaptations (Shichida et al., 2013). With the degeneration of their other senses (e.g., vision and hearing), snakes have developed a keen sense of smell. Together with their diverse habitats, snakes thus provide an excellent opportunity to explore the molecular basis and expression features of olfactory systems in vertebrates from different habitats (Lillywhite, 2014). In this study, we generated RNA-seq data from MOS and AOS tissues of aquatic and non-aquatic snakes and constructed a co-expression network related to habitat type. Results revealed unique gene expression profiles associated with environmental adaptation, especially key expression networks of aquatic adaptation. Given the lack of relevant published data, our findings provide an important resource for exploring the adaptation of vertebrate olfactory systems to special habitats.

Our results showed that both aquatic and non-aquatic MOS-related modules were involved in synapse and neuron-related terms, indicating similar functions and the key role of synapses in the MOS. In the non-aquatic MOS module, seven genes participated in the regulation of synapse formation and olfactory neuron maturation and homeostasis. *PTPRO* is implicated in synapse growth and guidance during embryonic development (Kotani et al., 2010). *ADCY3* plays an indispensable role in postnatal maturation of olfactory sensory neurons and the absence of *ADCY3* leads to cumulative defects in olfactory sensory neuronsmaturation (Zhang et al., 2017). *CFTR* is localized in microvillar cells of the olfactory epithelium and regulates microvillar cells to maintain neuronal tissue homeostasis (Pfister et al., 2015). In the aquatic MOS module, synapse-related terms were enriched in different genes, including *GABRA2*, *OPHN1*, *RELN*, *TBX21*, *IL1RAPL1*, *SHC4*, *KIRREL3*, *SYT2*, and *SYT10*. *GABRA2*, *OPHN1*, and *RELN* are involved in the regulation of inhibitory neuronal formation, while the six other genes are involved in the regulation of neuronal maturation. *RELN* was also identified as a highly expressed hub gene in the aquatic MOS co-expression network, indicating its vital role in the MOS of aquatic snakes. In mice, *RELN* knockout results in disruption of the granular cell layer (Martin-Lopez et al., 2012). Granular cells inhibit mitral cells (MCs) through their dendrodendritic reciprocal connections, and inhibition of MCs can weaken odor representation and enhance the signal-to-noise ratio (Isaacson and Strowbridge, 1998; Koulakov and Rinberg, 2011). Therefore, *RELN* likely plays a vital role in aquatic snake MOS and may facilitate adaptation to aquatic environments by regulating the granular cell layer. Additionally, inhibitory neuron activation can reduce the excitability of MCs and increase MCs pattern separation, which enables the olfactory bulb to disambiguate sensory stimuli with overlapping features (Gschwend et al., 2015). Regulation of the maturation and formation of inhibitory neurons is regulated by *OPHN1* and *GABRA2* (Pallotto et al., 2012; Redolfi et al., 2016), which showed higher expression in the aquatic snakes. The nasal cavity in snakes bears a rich sensory epithelium that is sensitive to air-borne volatile chemicals (Lillywhite, 2014). In aquatic snakes, however, degradation of the MOS and large-scale reduction in olfactory receptors suggest degeneration of the aquatic MOS, which could be interpreted as an adaptation to environmental media (Kishida and Hikida, 2010; Kishida et al., 2019; Peng et al., 2020; Li et al., 2021). Our results suggest that aquatic snakes may increase neuronal pattern separation *via* the inhibitory neurons suppressing MCs to eliminate overlapping information from olfactory stimuli, thereby enabling the acquisition of valuable information within complex water environments. Note, in addition to the habitat types, diet may also affect the expression pattern of MOS, which may explain the clustering pattern of transcriptome data, i.e., in the MOS data aquatic snakes clustered in a group and could be clearly distinguished from *P. menglaensis* (Danaisawadi et al., 2016). *P. menglaensis* specializes in eating snails and the diet is unique to other snakes (Auer et al., 2020). Therefore, more species are required for exploring the effect of multifactors on olfactory system gene expression patterns in subsequent studies.

Changes in receptor molecule copy number may be related to animal evolution, with such changes possibly caused by habitat alteration and adaptation (Shichida et al., 2013). Previous comparative genomics analysis of seven vertebrates from terrestrial and aquatic environments found that the ratio of *V1R* to *V2R* genes increased during vertebrate evolution from water to land (Shi and Zhang, 2007). Here, in the aquatic AOS modules, *TRPC2* and 22 receptor genes (21 *V2Rs* and *GPR180*) were enriched in the response to pheromone GO term. Among these genes, *TRPC2* and *V2R26_10* were identified as hub genes and were highly expressed with 11 other *V2R* transcripts in the aquatic snake AOS. *V2R* genes are putative pheromone receptors and are related to Ca^2+^-sensing receptors and metabotropic glutamate receptors (Halpern, 2003). In aquatic species, *V2R* genes are often found with a large number of copies. For example, previous research on two true sea snakes suggested that considerable expansion of the underwater olfaction-related *V2R* gene family likely occurred in ancestral species of the *Hydrophiini* clade, with >1,000 copies found in *Hydrophis cyanocinctus* and *Hydrophis curtus*, but only 389 found in the common viper (Li et al., 2021). Moreover, the aquatic species *Xenopus tropicalis* contains 330 *V2R* genes, while the semiaquatic red-legged salamander only contains 34 *V2R* genes (Ji et al., 2009; Kiemnec-Tyburczy et al., 2011; Herrel et al., 2014). These results suggest that aquatic snakes may have enhanced ability to bind water-soluble odor molecules in aqueous media by increased copy number and expression of *V2Rs*.

*TRPC2* may also play a fundamental role in aquatic adaptation. *TRPC2* drives the process of Na^+^ and Ca^2+^ influx-induced depolarization (Kaupp, 2010). In addition, *TRPC2* is pseudogenized in the aquatic fin whale and semiaquatic harbor seal and river otter, which lack AOS, but is functionally intact in California sea lions, which possess AOS (Yu et al., 2010). In the current study, a subnetwork containing *TRPC2* and *V2R26_10* was constructed, which showed the co-expression of genes involved in neuronal differentiation (e.g., *OTX2*, *LHX2*, *NEUROG1*, *INSM1*, and *FOXG1*). Odor perception is caused by combining odor molecules with different receptors in sensory neurons (Ubeda-Banon et al., 2011). *LHX2* is expressed in the olfactory epithelium and guides the differentiation of progenitor cells into a thousand neuronal subpopulations, each expressing a unique receptor gene (Kolterud et al., 2004). *Insm1* is localized in the olfactory epithelium and promotes the transition of progenitors from apical and proliferative to basal, terminal, and neurogenic (Rosenbaum et al., 2011). Overall, aquatic snake adaptations to the water environment depended on changes in receptors and ion channels. Furthermore, the co-expression network with the *TRPC2* hub gene played an essential role in adaptation, with *TRPC2* potentially exerting its actions by regulating the five genes involved in neural differentiation.

In the current study, we comprehensively analyzed the olfactory system transcriptome of four snakes, considering different habitat conditions. Our findings indicated that the gene expression patterns of the olfactory systems differed between aquatic and non-aquatic snakes. In the MOS of aquatic snakes, *RELN* may improve the identification of valuable chemical information *via* the inhibitory neurons suppressing MCs to increase neuronal pattern separation, which may be in favour of aquatic adaptation. In the AOS of aquatic snakes, high expression of the *TRPC2*, together with vomeronasal receptor genes, may intensify their ability to bind water-soluble odor molecules to promote response to the aquatic environment. Our results provide a basis for future studies on olfactory systems and environmental adaptation in snakes.

## Data Availability

The data presented in the study are deposited in the China National GeneBank (CNGB) repository, accession number CNP0002425.

## Footnotes

1
**^** https://ftp.uniprot.org/pub/databases/uniprot/

2
**^** https://wego.genomics.cn/

3
**^** http://revigo.irb.hr/
