# Supplementary material for: Transcriptome Analysis Reveals Olfactory System Expression Characteristics of Aquatic Snakes
Source: Front Genet. 2022 Jan 25;13:825974. doi: 10.3389/fgene.2022.825974 (PMC8829814; doi:10.3389/fgene.2022.825974)
Supplement: Supplementary file 1 [file DataSheet1.zip › Supplementary Figure.docx]

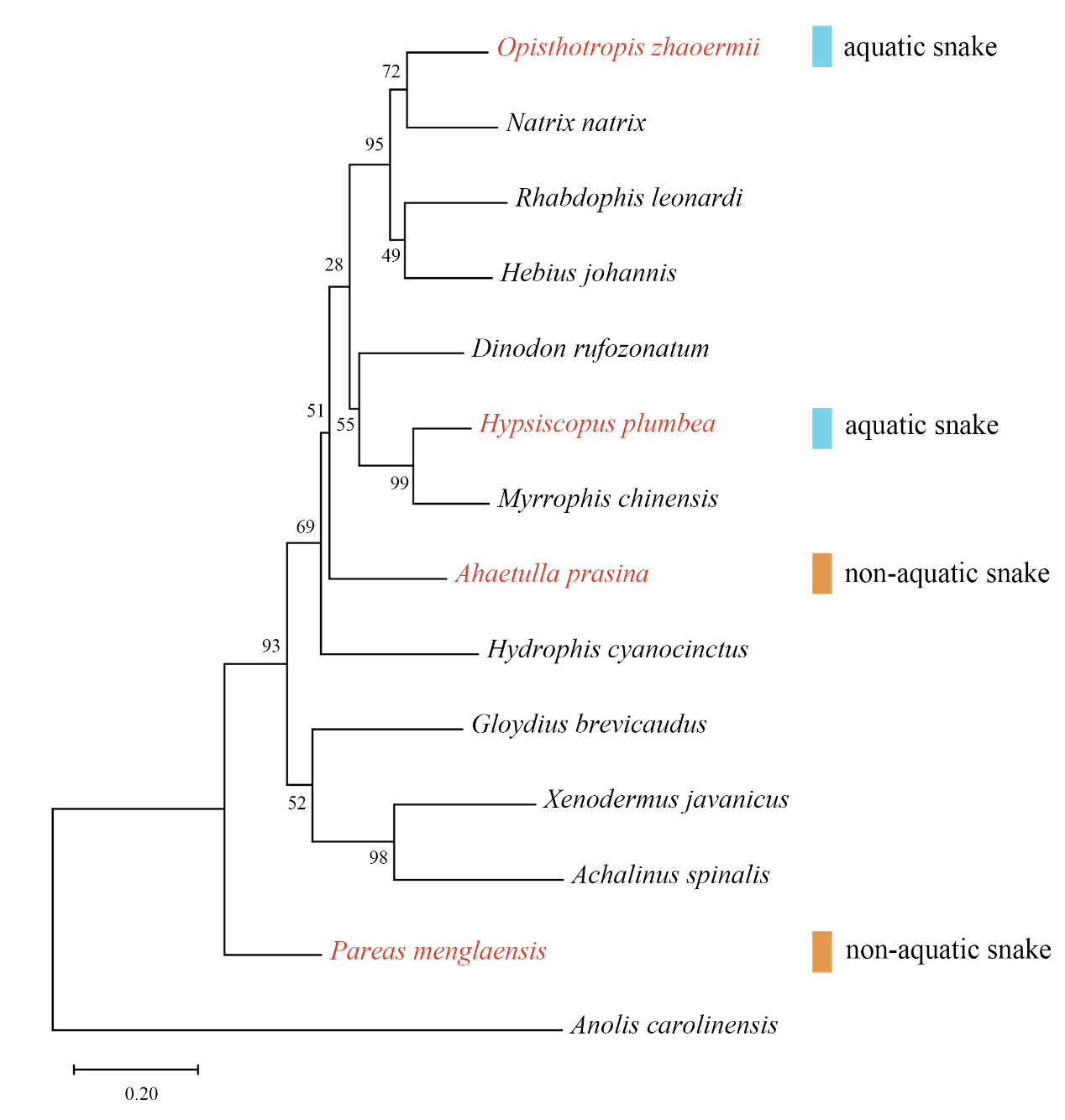


**Supplementary Figure 1.** Phylogenetic analysis of four species. The species selected for the experiment are marked red, and the blue squares represent aquatic snakes and the yellow squares represent non-aquatic snakes. Anolis carolinensis was selected as the outgroup for phylogenetic analyses, and 14 available cyt b sequences were downloaded from Genbank to construct the phylogenetic tree by using MEGA-X. The species and GenBank accession No. are as follows: *Ahaetulla prasine* (LC105637), *Opisthotropis zhaoermii* (MG012799), *Hypsiscopus plumbea* (NC_010200), *Pareas menglaensis* (MK135114), *Anolis carolinensis* (EU747728), *Achalinus spinalis* (NC_032084), *Dinodon rufozonatum* (KC733196), *Gloydius brevicaudus* (JQ687497), *Hydrophis cyanocinctus* (NC_046795), *Hebius johannis* (KJ685708), *Myrrophis chinensis* (MT802727), *Natrix natrix* (HF680010), *Rhabdophis leonardi* (KF800933), *Xenodermus javanicus* (AY425810).


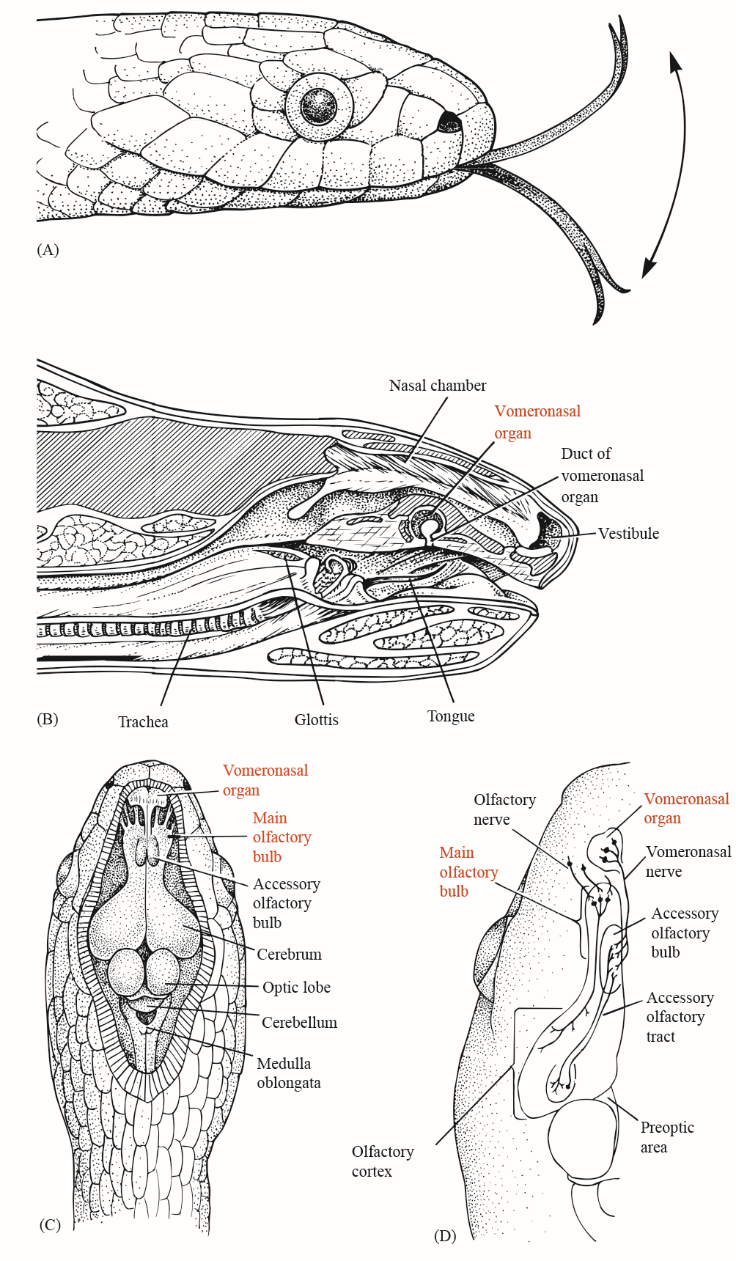


**Supplementary Figure 2.** Schematic diagram of olfactory tissue. Source: The picture adapted from Kardong K.V. (2012).


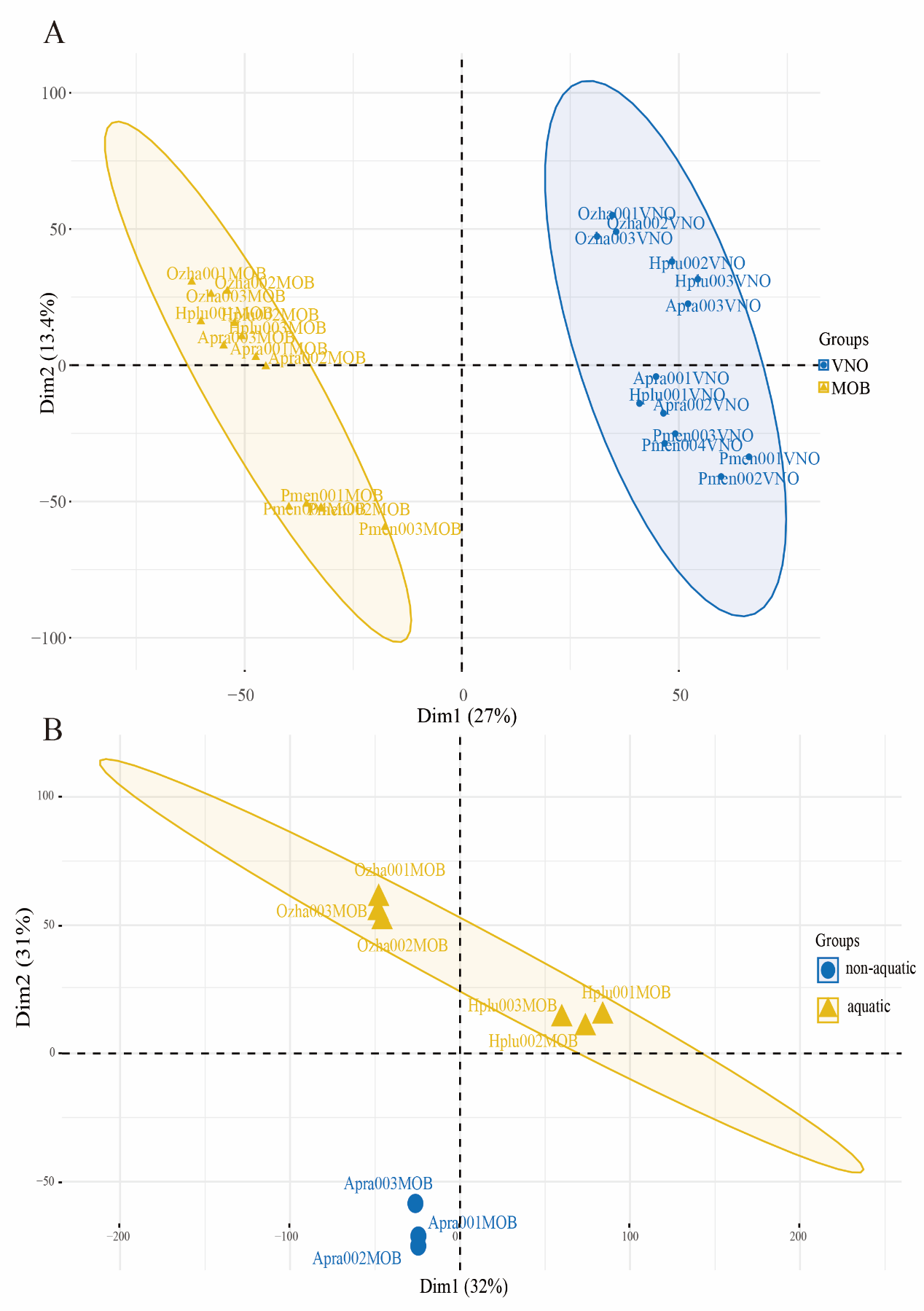


**Supplementary Figure 3.** Principal component analysis (PCA). (A) PCA of 26 samples. (B) The MOS samples of *Ahaetulla prasine, Hypsiscopus plumbea* and *Opisthotropis zhaoermii* were performed PCA.


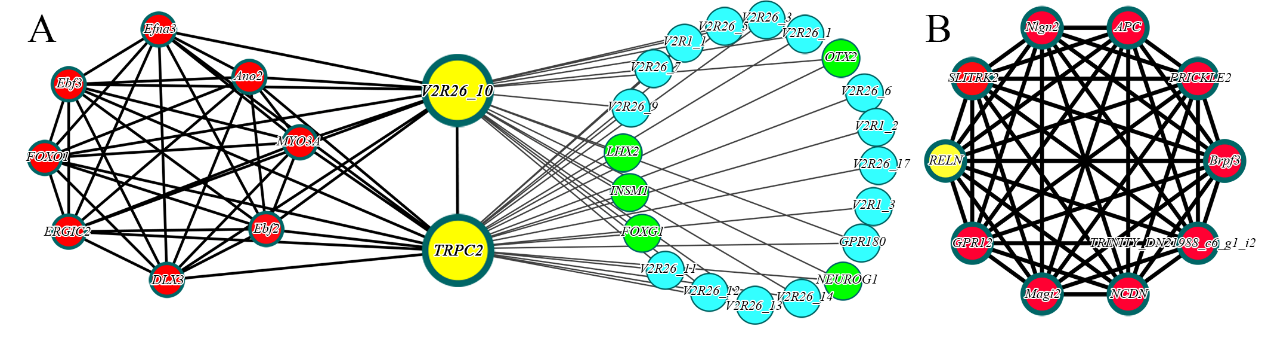


**Supplementary Figure 4.** Co-expression network of hub genes in the aquatic MOS and AOS modules. The genes in the red circle are hub genes. The genes in the yellow circle are olfactory-related hub genes The genes in the blue circle are vomeronasal receptor genes. And the genes in the cyan circle are associated with neural development. (A) Co-expression network of hub genes (left) and the subnetwork of TRPC2 and V2R26_10 (right) in the aquatic AOS module. (B) Co-expression network of hub genes in the aquatic MOS module.
